# A Novel Method for Volumetric MRI Response Assessment of Enhancing Brain Tumors

**DOI:** 10.1371/journal.pone.0016031

**Published:** 2011-01-26

**Authors:** Charles W. Kanaly, Dale Ding, Ankit I. Mehta, Anthony F. Waller, Ian Crocker, Annick Desjardins, David A. Reardon, Allan H. Friedman, Darell D. Bigner, John H. Sampson

**Affiliations:** 1 Division of Neurosurgery, Department of Surgery, Duke University Medical Center, Durham, North Carolina, United States of America; 2 Department of Neurosurgery, The University of Virginia, Charlottesville, Virginia, United States of America; 3 Department of Radiation Oncology, Emory University, Atlanta, Georgia, United States of America; 4 Department of Neurology, Duke University Medical Center, Durham, North Carolina, United States of America; 5 Department of Pathology, Duke University Medical Center, Durham, North Carolina, United States of America; The University of Chicago, United States

## Abstract

Current radiographic response criteria for brain tumors have difficulty describing changes surrounding postoperative resection cavities. Volumetric techniques may offer improved assessment, however usually are time-consuming, subjective and require expert opinion and specialized magnetic resonance imaging (MRI) sequences. We describe the application of a novel volumetric software algorithm that is nearly fully automated and uses standard T1 pre- and post-contrast MRI sequences. T1-weighted pre- and post-contrast images are automatically fused and normalized. The tumor region of interest is grossly outlined by the user. An atlas of the nasal mucosa is automatically detected and used to normalize levels of enhancement. The volume of enhancing tumor is then automatically calculated. We tested the ability of our method to calculate enhancing tumor volume with resection cavity collapse and when the enhancing tumor is obscured by subacute blood in a resection cavity. To determine variability in results, we compared narrowly-defined tumor regions with tumor regions that include adjacent meningeal enhancement and also compared different contrast enhancement threshold levels used for the automatic calculation of enhancing tumor volume. Our method quantified enhancing tumor volume despite resection cavity collapse. It detected tumor volume increase in the midst of blood products that incorrectly caused decreased measurements by other techniques. Similar trends in volume changes across scans were seen with inclusion or exclusion of meningeal enhancement and despite different automated thresholds for tissue enhancement. Our approach appears to overcome many of the challenges with response assessment of enhancing brain tumors and warrants further examination and validation.

## Introduction

Assessing radiographic response in patients with brain tumors can be challenging, especially since they often contain large cysts or resection cavities and may contain postoperative blood products that create magnetic resonance imaging (MRI) signal changes similar to contrast-enhancement. Resection cavities also frequently have irregular shapes with satellite lesions and small amounts of postoperative residual rim enhancement which are difficult to quantify. In addition, over time the cavities can collapse which dramatically alters the size and configuration of these irregular enhancing areas.[1], [2], [3] Thus changes in actual tumor volume are difficult to describe with traditional measurement techniques.

**Figure 1:**
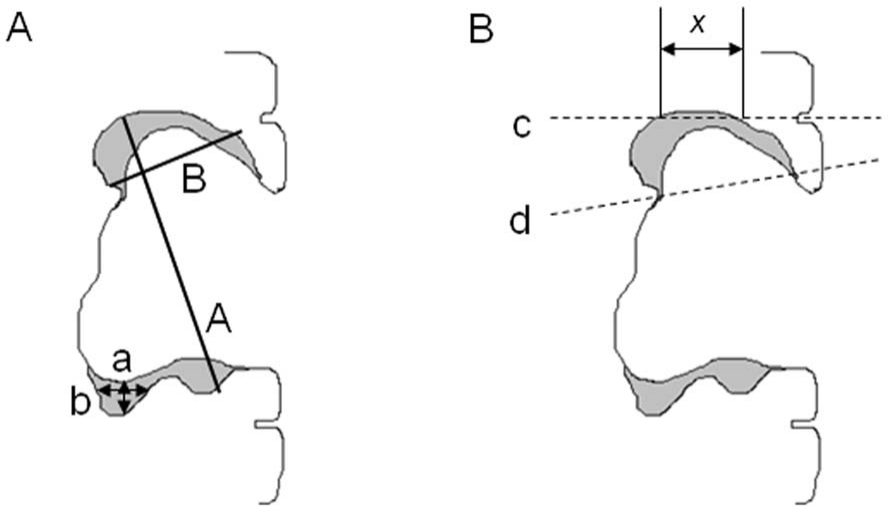
Traditional Non-Volumetric Measurements do not Adequately Describe Residual Enhancement in Surgical Resection Cavities. A) This schematic resection cavity has residual rim enhancement in gray. RECIST criteria measurement ‘A’ or ‘a’ or ‘b’ or Macdonald criteria measurement ‘A*B’ or ‘a*b’ would not adequately describe residual tumor volume and additional tumor growth around the rim or collapse of the resection cavity may be over- or under-interpreted. B) Differences in axial slice acquisition also impact measurements made by traditional criteria more than volumentric measurements. One scan could obtain axial slice ‘c’ with enhancing tumor measurement ‘x’ but a subsequent scan in the same patient could obtain axial slice ‘d’, causing an incorrect assessment of tumor response.

The most commonly used methods to determine treatment responses in brain tumors are the Macdonald criteria[4] and the Response Evaluation Criteria in Solid Tumors (RECIST) criteria [5]. While the Macdonald criteria incorporate two-dimensional (2D) measurements with steroid dosing and the patients' neurological examinations, the more recent RECIST criteria evaluate tumor response based on measurement of the longest one-dimensional (1D) diameter. The radiologic assessment for neuro-oncology (RANO) criteria[6] published recently represent a modification of the Macdonald criteria that include guidelines for assessing response in the context of pseudoprogression that can confound MRI interpretation after combined TMZ chemoradiotherapy[7]. The RANO criteria also specify the inclusion of non-enhancing tumor burden for the assessment of response that has become relevant in the modern era with the widespread use of anti-angiogenic agents. Despite their ease of use and widespread application, the Macdonald, RANO and RECIST criteria cannot accurately describe the amount of residual tumor in surgical resection cavities (Fig. 1).[2], [8] The initial paper by Macdonald *et al.* even acknowledges that their criteria should not be applied to resection cavities, and the RECIST criteria consider all lesions that are either less than 1 cm or cystic to be unmeasurable, which would likely exclude rim enhancement around a postoperative resection cavity.[9] Because of the difficulty applying these existing methods to postoperative patients, there has been conflicting evidence that response assessments made based on these methods are related to time to progression.[10], [11], [12] Progression free survival is nonetheless becoming a more frequently used endpoint in clinical trials[11], [12] and therefore the need is increasing for improved response assessment techniques.

Other authors have suggested modifications to these criteria including measuring across cystic areas or resection cavities and then subtracting a measurement of the cystic areas.[13] A more complicated scheme used in a major multicenter phase III clinical trial involves the summation of 2D areas of all tumor nodules with an estimate of the 2D area of rim enhancement, which is calculated by multiplying a curvilinear length of the resection cavity multiplied by the width. Rim enhancement in this trial is only considered measurable if the width is 5 mm or greater in width[14]. Despite their increased complexity, these modified techniques still do not entirely account for small changes that occur in resection cavities, and alternatively many of these issues can be overcome with volumetric measurements that can assess the entire tumor burden.

Volumetric techniques are currently feasible with modern three-dimensional reconstruction stations and intraoperative navigation systems, but mostly require experienced operators to manually outline the tumor volume and then perform the analysis. Since they depend on the user to outline tumor volume manually, there can be considerable inta- and inter-user variability in the inclusion of rim-enhancing resection cavities, blood or other bright lesions in the resection cavity, and there can be difficulties determining where the tumor margin ends and normal tissue begins. Furthermore, these methods can be influenced by differences in slice acquisition as well as the timing and dose of contrast boluses.

We describe a novel, nearly fully automated software algorithm that measures enhancing tumor volume and runs on a standard laptop computer using standard DICOM images of pre- and post- contrast MRI scans. In this study, we demonstrate that our method is able to measure tumor volume changes despite resection cavity collapse, while traditional 1D and 2D techniques are incorrectly influenced by the overall resection cavity configuration. We are also able to determine the presence of enhancing tumor when it is obscured by intrinsically bright T1 objects, such as subacute hemorrhage in a resection cavity. To demonstrate the two extremes of inter-user variability, we show similar trends in our results when the enhancing volume is narrowly defined or generous and includes the adjacent enhancing meninges. We also determine that the changes in volume calculations that occur with different threshold levels for determining tissue enhancement are minimal.

## Materials and Methods

**Figure 2:**
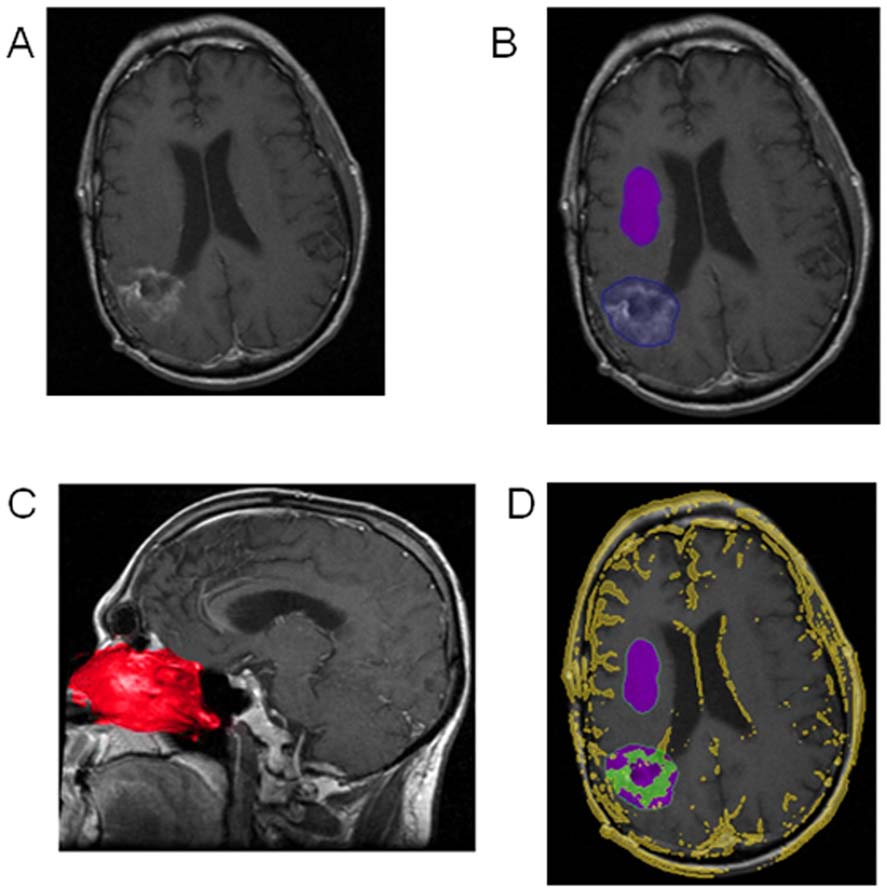
Automated Assessment of Enhancing Tumor Volume. A) T1-weighted post-contrast axial images are automatically fused with the pre-contrast sequences. B) The tumor region of interest (blue area) and nearby normal brain (purple area) are outlined roughly by hand. C) The enhancing nasal mucosa region is automatically detected with a built-in anatomic atlas (red area) and serves as a threshold for enhancement. D) Tissue that is present on the post-contrast images but not the pre-contrast that is above the enhancement threshold appears in yellow. This includes enhancing tissue such as vasculature, tumor, and superficial structures. Enhancing tumor volume is defined as the green area within the manually-defined blue tumor region of interest.

Standard DICOM images of T1-weighted magnetic resonance imaging (MRI) axial images of the brain before and after contrast were imported into a standard laptop computer installed with our novel volumetric assessment software. The computer program automatically fuse pre and post contrast images (Fig. 2A). As depicted in Fig. 2B, the user grossly outlined the tumor region of interest in the T1-weighted axial post-contrast scan and arbitrarily outlined a region of normal brain parenchyma, not including vessels, ventricles, sulci, or other cerebrospinal fluid (CSF) spaces. The computer program automatically analyzed the outlined normal brain parenchyma to compensate for variability in scan brightness and lack of standardized pixel values in MRI scans. For studies involving meningeal enhancement, the operator intentionally grossly outlined the tumor region with and without including the adjacent meninges.

The program automatically detected the nasal mucosa based on an anatomic atlas (Fig. 2C). It automatically calculated enhancing tumor volume from within the grossly defined region of interest, as depicted in Fig. 2D, using a rigorous computational algorithm that can be briefly described as follows. Within the outlined normal region, our program calculated the subtracted pixel values between pre- and post-contrast scans. The mode of these subtracted values was determined and used as a correction factor for all subsequent data analyses. The pixel values within the tumor region and the nasal mucosa region were subtracted between pre- and post-contrast scans, and then this calculated correction factor was also subtracted, to generate corrected tumor values and corrected mucosa values. The top 5% of the corrected nasal mucosa values derived from the nasal mucosa atlas were excluded to avoid error, and then the threshold for enhancement was set to be 25% of this remaining maximum value. This number was subsequently used as the threshold above which tissue was defined to be enhancing. For the studies involving different threshold levels, 25%, and 40% were used as the different experimental threshold values. The number of pixels in the tumor region of interest above the enhancement cut-off was calculated to determine the enhancing tumor volume. These calculations are all performed automatically once the regions of interest are grossly outlined by the user.

## Results

### Detection of Enhancing Tumor with Resection Cavity Collapse

**Figure 3:**
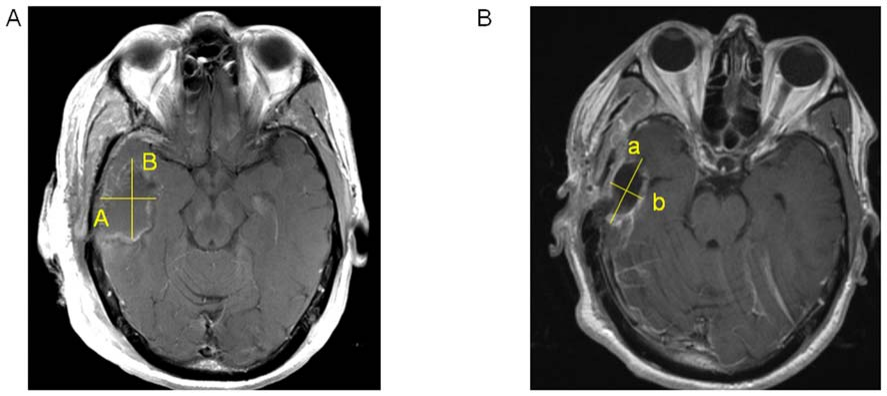
Detection of Enhancing Tumor Volume Despite Resection Cavity Collapse. A) T1-weighted post-contrast axial image showing a resection cavity with rim enhancement. RECIST measurement would be A and Macdonald measurement would be “A * B”. B) T1-weighted post-contrast axial image showing the same patient 3 months postoperatively who had collapse of his resection cavity. RECIST measurement would be “a” and Macdonald measurement would be “a * b”, both of which would be smaller than the measurements from the initial scan above, but this change would be describing only the resection cavity configuration and not the underlying tumor burden.

Traditional methods struggle to determine changes in enhancing tumor when the configuration of the resection cavity changes. Fig. 3A demonstrates a single axial slice from an MRI of a postoperative resection cavity with rim enhancement. Using the RECIST criteria, measurement would be 4.3 cm (“A”). Similarly, using the Macdonald criteria, the measurement would be 4.3 cm * 3.2 cm = 13.8 cm^2^ (“A” * “B”). Volumetric measurement of the area around the resection cavity with our method identifies 1.26 cm^3^ of enhancing tumor, which intuitively appears to quantify the amount of residual tumor more accurately. Moreover, it allows more accurate comparisons with follow-up scans. For example, Fig. 3B demonstrates collapse of this resection cavity, and both RECIST measurement (“a”  = 3.7 cm) and the Macdonald measurement (“a * b,”  = 3.7 * 1.7 = 6.29 cm^2^) are smaller due to this cavity collapse, which appears counter-intuitive. Our volumetric method, however, identifies 5.58 cm^3^ of enhancing tumor indicating that the tumor burden had actually increased. However, Macdonald and RECIST measurements which included changes in the resection cavity configuration, assess overall tumor enhancement as decreasing despite an increase in rim enhancement. Of note, the patient continued to progress over the next four months with an increase in enhancement volume to 9.82 cm^3^,Tumor progression was also confirmed with a subsequent biopsy in this case.

### Subacute Blood

**Figure 4:**
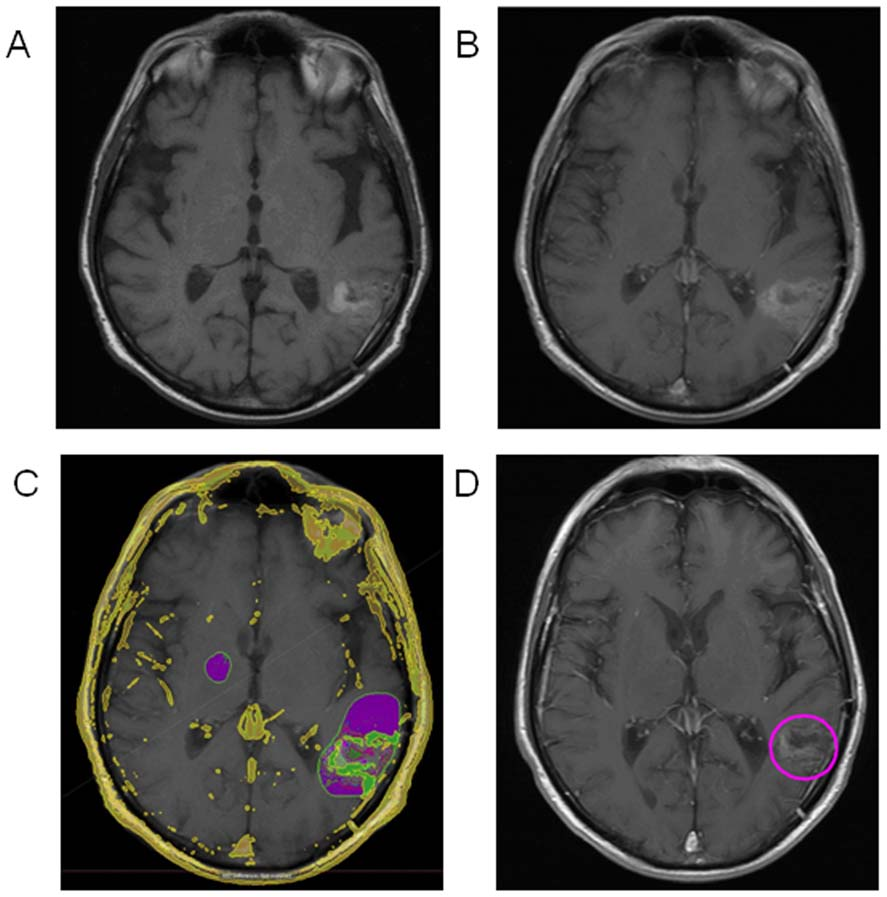
Detection of Enhancement that is Obscured by Blood Products. A) Uncontrasted T1-weighted axial image showing resection cavity blood products (bright on T1). B) T1-weighted post-contrast axial image showing the difficulty in determining residual enhancing tumor. C) Our volumetric analysis is able to detect the obscured enhancing tumor tissue (shown in green). D) T1-weighted post-contrast axial image at 2.5 months later after the blood has resolved verifying the underlying enhancing tumor volume.

By comparing between pre- and post-contrast scans and using nasal mucosa enhancement as a reference, our volumetric program is able to subtract intrinsically bright T1 signal so that elements that are bright on both scans (*i.e.* blood products in a resection cavity) can be differentiated from enhancing tumor that is only bright in the post-contrast scan. Figs. 4A and 4B show an example where the intrinsically bright T1 signal of subacute blood has made it difficult to determine the presence of enhancing tumor on the post-contrast scan. Our volumetric method was able to successfully detect 3.06 cc of residual tumor enhancement despite the presence of intrinsically high T1 signal, as shown in Fig. 4C. RECIST measurement was 3.6 cm and Macdonald measurement was 8.64 cm^2^. The enhancing tumor volume calculated by our methods corresponds well to the true residual enhancing tumor, as confirmed on a subsequent MRI performed 2.5 months later in this patient after the subacute blood had resolved to reveal a residual enhancing tumor of 4.48 cc (Fig. 4D).

### Variability in User Defined Tumor Region

**Figure 5:**
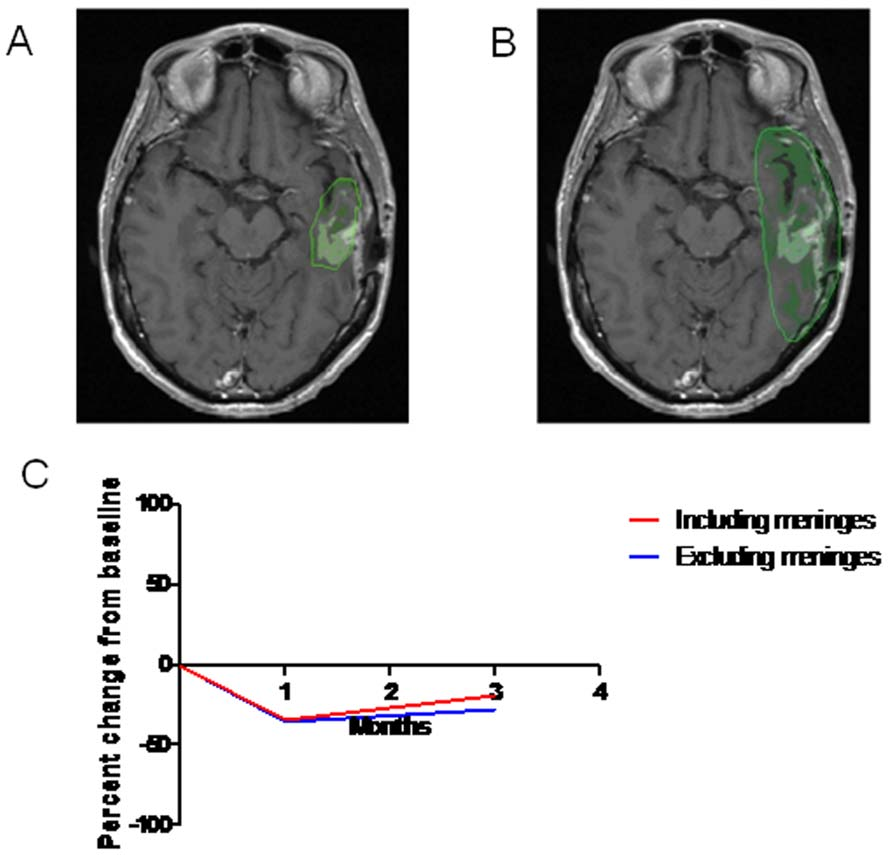
Effect of Inter-observer Differences in Definition of Tumor Volume. A) Axial T1-weighted post-contrast image showing a limited user-defined tumor region of interest. B) The same axial image now showing a large user-defined tumor region of interest that encompasses the meningeal enhancement. C) While including the meninges increases the enhancing volume, similar trends in changes of volume over time are seen.

Most volumetric techniques are hampered by the need for expert opinion to distinguish true enhancing tumor from adjacent normal brain and postoperative changes such as enhancing meningeal scar. Similarly, a potential source of variability in the use of any volumetric method is the amount of adjacent enhancement (including meningeal scarring, blood vessels, choroid plexus, etc.) the user includes when grossly outlining the region of interest. As Figs. 5A and 5B demonstrate, when a generous tumor region of interest including surrounding meningeal enhancement is outlined, the total calculated enhancing volume is greater than when a narrowly defined tumor region is outlined. However, a benefit of our method is that sequential scans are fused together by the program so that the tumor region of interest grossly outlined on the initial patient scan is directly transferred to subsequent scans for that patient. Enhancing volume calculations of serial scans both with and without the adjacent meningeal enhancement were performed and Fig. 5C shows that there is little difference in the change between scans regardless of technique to define the tumor region of interest.

### Variability in Enhancement Threshold

**Figure 6:**
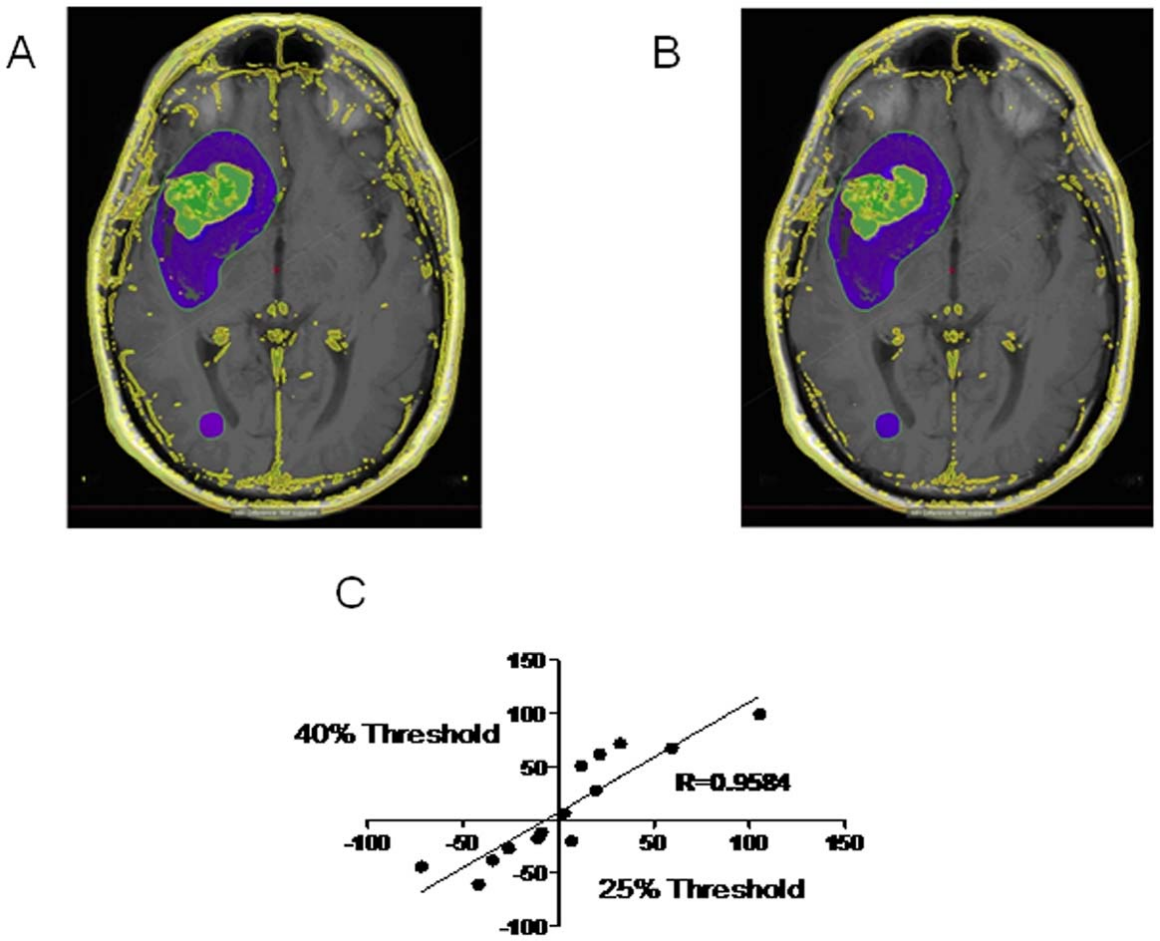
Effect of Different Enhancement Thresholds. A) Axial T1-weighted post-contrast image after volumetric analysis has been performed which shows in green the detected enhancing tumor volume using a 25% threshold level. B) Detected enhancing tumor volume using a 40% threshold level. C) While increasing the threshold decreases the calculated tumor volume, the volumes across different threshold levels are highly correlated.

Our semi-automated volumetric method uses a pre-defined percent threshold of the enhancing nasal mucosa in each patient scan for identification of enhancing tissue within the tumor region of interest. Increasing the percent threshold will decrease the calculated enhancing volume, while decreasing the threshold will increase the calculated volume, as graphically depicted in Figs. 6A–B. Fig. 6C shows the results of enhancing volume analysis with different thresholds in serial scans of patients. Comparison between a 40% threshold with a 25% threshold yielded a high correlation (r = 0.96; P<0.0001). Therefore, when the defined threshold level is maintained consistently through serial scans, the results are highly correlated regardless of the specific threshold level.

## Discussion

Our volumetric method demonstrates promise in the response assessment of enhancing brain tumors. It can account for enhancing tumor despite expected postoperative collapse of the resection cavity and is able to detect enhancing tumor in the midst of intrinsically bright T1 signal, such as in postoperative resection cavities with a small amount of hemorrhage. These situations frequently occur in brain tumor patients after surgical resection and have been difficult to describe with traditional response assessment methods.

Technical differences, such as contrast dose and gantry angle, for serial MRI scans can lead to the false appearance of changes in the amount of tumor enhancement. Our method overcomes these problems by using image fusion to eliminate differences from slice acquisition, and it uses a cutoff for enhancement that is calculated for each scan to minimize the importance of variations in contrast bolus and enhancing signal intensity between different MRI scans.

Since our program runs on a laptop computer and analyzes DICOM data directly from standard T1 pre- and post-contrasted sequences that patients typically bring to clinic on a CD, widespread adoption of our method would be easier than other techniques that require specialized MRI sequences or computer systems. For example, others have developed a parametric response map with perfusion MRI data to predict overall survival.[15] A significant amount of work has also been devoted to adapting diffusion MRI imaging for the assessment of tumor response[16], [17], [18], [19]. These and similar techniques are hindered in their acceptance since these specialized sequences may not be routinely obtained.

It is expected that the increased automation of our approach should improve the reproducibility of calculated tumor volumes, but this still needs to be studied and validated prospectively. In support of this assumption, Schwartz *et al.* found that in CT assessment of solid tumors, techniques that employed increased automation obtained results that were more accurate and consistent than manual methods.[20] Other studies using automated CT volumetric methods in pulmonary tumors suggest superiority when compared to manual RECIST measurements. [21], [22] For gliomas, Sorensen *et al.* found that a computer-assisted perimeter method of volume calculation produced less inter- and intra-user variability than a manual volumetric calculation that used diameter measurements.[13] In addition to increased automation, our method specifically detects only enhancing tissue. Other semi-automated tumor assessment methods, including automatic segmentation methods that use fuzzy clustering and interactive watershed algorithms, do not take into account tumor enhancement specifically.[23], [24], [25]

Our results suggest that there would be limited variability in results with different users of our method, since similar trends in volume change were seen with both inclusion or exclusion of the adjacent meningeal enhancement. Future studies will be directed at examination of inter-user variability with our method. Generally, volumetric measurements are expected to have less measurement variability than 1D or 2D methods, thereby improving repeatability and possibly decreasing the sample size needed in studies to ascertain treatment effects.[2] By accounting for the entire tumor burden, it is expected that volumetric measurement should allow for the detection of tumor response or progression sooner than Macdonald and RECIST criteria. This should substantially reduce intra-observer variability. It is likely that these two different user techniques will not substantially impact response assessment as tumor response criteria are based on percent changes between scans and not absolute measurements. In support of these assumptions, Dempsey *et al.* found that volumetric measurements, and not 1D or 2D methods, were predictive of survival.[26] Numerous other studies have been performed comparing 1D, 2D and volumetric measurements, with many showing good agreement between all three methods in classifying response and predicting survival.[27], [28] Comparisons made by Warren *et al.* in pediatric brain tumor patients found higher concordance between 1D and 2D methods than either compared with manual volumetric measurements, and although all three techniques had high concordance in detecting partial responses, this was not true for minor responses or tumor progression, and there was significant variability across measurements in estimating time to disease progression.[29] CT-based volumetric assessment of solid tumors has shown varying degrees of correlation with 1D and 2D methods.[30], [31] Formal comparison of our method with traditional assessment techniques is planned.

An important difficulty with measuring enhancing brain tumors on MRI is that there is no quantitative cutoff for tissue enhancement on MRI. There have been a few previous descriptions of a formulaic determination of an enhancement threshold based on the initial peak enhancing signal increase, but this has not been widely accepted.[32], [33] Many previous methods have simply utilized expert opinion to select the enhancing tissue according to their best judgment, however this technique invites significant subjective error, as different experts may have different opinions. Our method attempts to address this inherent limitation of MRI scans and still minimize subjectivity by increasing computer automation and calculating an internal threshold for tissue enhancement. The 25% threshold level we have adopted is based on expert radiologist opinion determining which level best corresponds to their judgement of enhancing tissue. Use of a standard threshold has precedence in other fields. With positron emission tomography (PET) scans, the detected intensity tapers off over distance from the source, so it is difficult to delineate precisely where the intensity is no longer apparent. A number of different methods have been attempted to estimate the tumor region of interest, and a set 40% threshold of either the source-to-background (S/B) ratio or of the maximum standardized uptake value (SUV) are commonly advocated techniques.[34], [35] Our results show a high degree of correlation between different threshold levels however, which supports the validity of this technique but also suggests that the specific threshold level chosen is not critical as long as the same level is used consistently.

Despite these advantages, our novel volumetric approach is based on the measurement of tissue enhancement, and therefore will not accurately quantify tumor burden if there is a significant amount of non-enhancing tumor. Even in enhancing tumors, there has recently been increasing use of anti-angiogenic agents such as bevacizumab that normalize vasculature and decrease enhancement leading to potential over-interpretations.[36], [37], [38] Other authors have noted that since enhancement can change due to radiation necrosis, pseudo-progression, steroid treatment, or pseudo-response, enhancement does not always reflect changes in the underlying tumor.[8] The RANO criteria were drafted to attempt to address these limitations[6]. Difficulty visualizing non-enhancing tumor burden is a problem for most proposed methods of assessing tumor response, and some authors have advocated that response criteria in these situations may have to be altered to include both radiologic changes and measurements of circulating biomarkers.[39] Unfortunately, these limitations are equally applicable to the Macdonald or RECIST criteria. The initial paper by Macdonald *et al.* even acknowledges that their criteria should not be applied to non-enhancing tumor.[4] As such, our method still represents an advance over current assessment techniques, and modification of our program to incorporate T2/FLAIR MRI sequences is currently being attempted to address this limitation.

Our novel volumetric method appears promising in the assessment of radiographic response for the majority of enhancing gliomas. Future studies will be needed to validate this method against other outcome assessment techniques, since subtle changes in tumor volume may not have clinical relevance. However our method offers the potential to characterize small enhancement changes that can occur over time due to a variety of factors including pseudoprogression, pseudoresponse, radiation necrosis, steroid treatment, and disease recurrence, as many of these imaging changes still lack detailed descriptions.[7], [40], [41], [42] Determination of the magnitude and time course of these different changes may lead to greater ability to distinguish between actual disease recurrence and other causes of enhancing volume change.
